# Vildagliptin‐induced ground‐glass nodules mimicking lung metastases in a cancer patient receiving *Lactobacillus* probiotic supplementation

**DOI:** 10.1111/1759-7714.13292

**Published:** 2020-01-06

**Authors:** Yasuhiro Tanaka, Hiroshi Soda, Yuichi Fukuda, Kenta Nio, Sawana Ono, Hiromi Tomono, Midori Shimada, Masataka Yoshida, Tatsuhiko Harada, Asuka Umemura, Keisuke Iwasaki, Hiroyuki Yamaguchi, Hiroshi Mukae

**Affiliations:** ^1^ Department of Respiratory Medicine Sasebo City General Hospital Nagasaki Japan; ^2^ Department of Medical Oncology Sasebo Kyosai Hospital Nagasaki Japan; ^3^ Department of Pathology Sasebo City General Hospital Nagasaki Japan; ^4^ Department of Respiratory Medicine Nagasaki University Graduate School of Biomedical Sciences Nagasaki Japan

**Keywords:** Bronchoalveolar lavage, interstitial pneumonia, probiotics

## Abstract

The association between gut microbiota and the lung immune system has been attracting increasing interest. Here, we report a case of pancreatic cancer in which the dipeptidyl peptidase‐4 inhibitor vildagliptin induced unusual manifestations of interstitial pneumonia, possibly under the influence of *Lactobacillus paraplantarum* probiotic supplementation. Chest computed tomography and positron emission tomography showed multiple ground‐glass nodules (GGNs) mimicking metastatic lung cancer. Transbronchial biopsy specimens showed mild fibrosis and infiltration of lymphocytes consisting of more CD4^+^ than CD8^+^ cells. The CD4^+^ cells did not include FOXP3^+^ regulatory T cells. Bronchoalveolar lavage confirmed lymphocytosis with a markedly increased CD4^+^/CD8^+^ ratio of 7.4. The nodules disappeared shortly after vildagliptin and probiotics were withheld. If unusual interstitial pneumonia is observed in some cancer patients, physicians should pay careful attention to their medication history, including probiotic supplements.

## Introduction

Cancer patients are reported to frequently take supplements including probiotics without prescription.^1^ Probiotics are micro‐organisms that could exert immunological effects by modulating a gut microbiota imbalance. Accumulated data have shown that gut microbiota contribute to the antitumor activity of immune checkpoint inhibitors.^2^, ^3^ The association between gut microbiota and the lung immune system may be critical in the treatment outcome of lung cancer.^4^ Moreover, various drugs are prone to cause interstitial pneumonia (IP) in lung cancer patients, at least in part through immunological mechanisms.^5^, ^6^ However, little is known about the effects of probiotics on drug‐induced IP in cancer patients. Here, we report the unusual manifestations of IP induced by the dipeptidyl peptidase‐4 (DPP‐4) inhibitor vildagliptin, possibly under the influence of *Lactobacillus paraplantarum* probiotic supplementation.

## Case report

**Figure 1:**
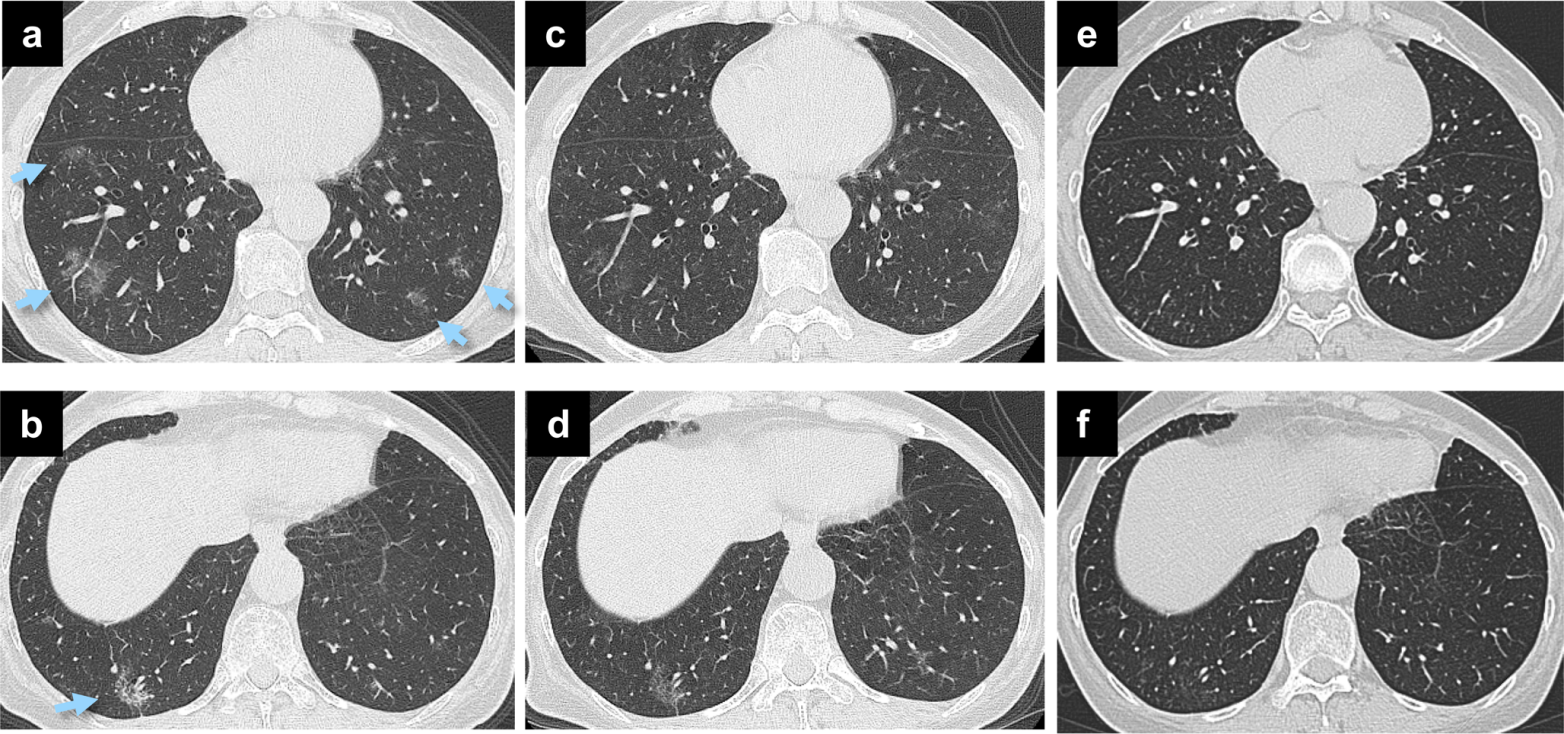
Chest computed tomography scans. Two months before the initial visit **(a)** multiple ground‐glass nodules (arrows) at the bilateral lower lobes and **(b)** the right S^10^ nodule with a pleural tag (arrow) were seen. Ten days after the cessation of vildagliptin and the *Lactobacillus paraplantarum* probiotic supplementation, **(c)** the multiple ground‐glass nodules had nearly disappeared and **(d)** the right S^10^ nodule had decreased in density. Four months later, **(e)** multiple ground‐glass nodules and **(f)** the right S^10^ nodule had entirely disappeared.

**Figure 2:**
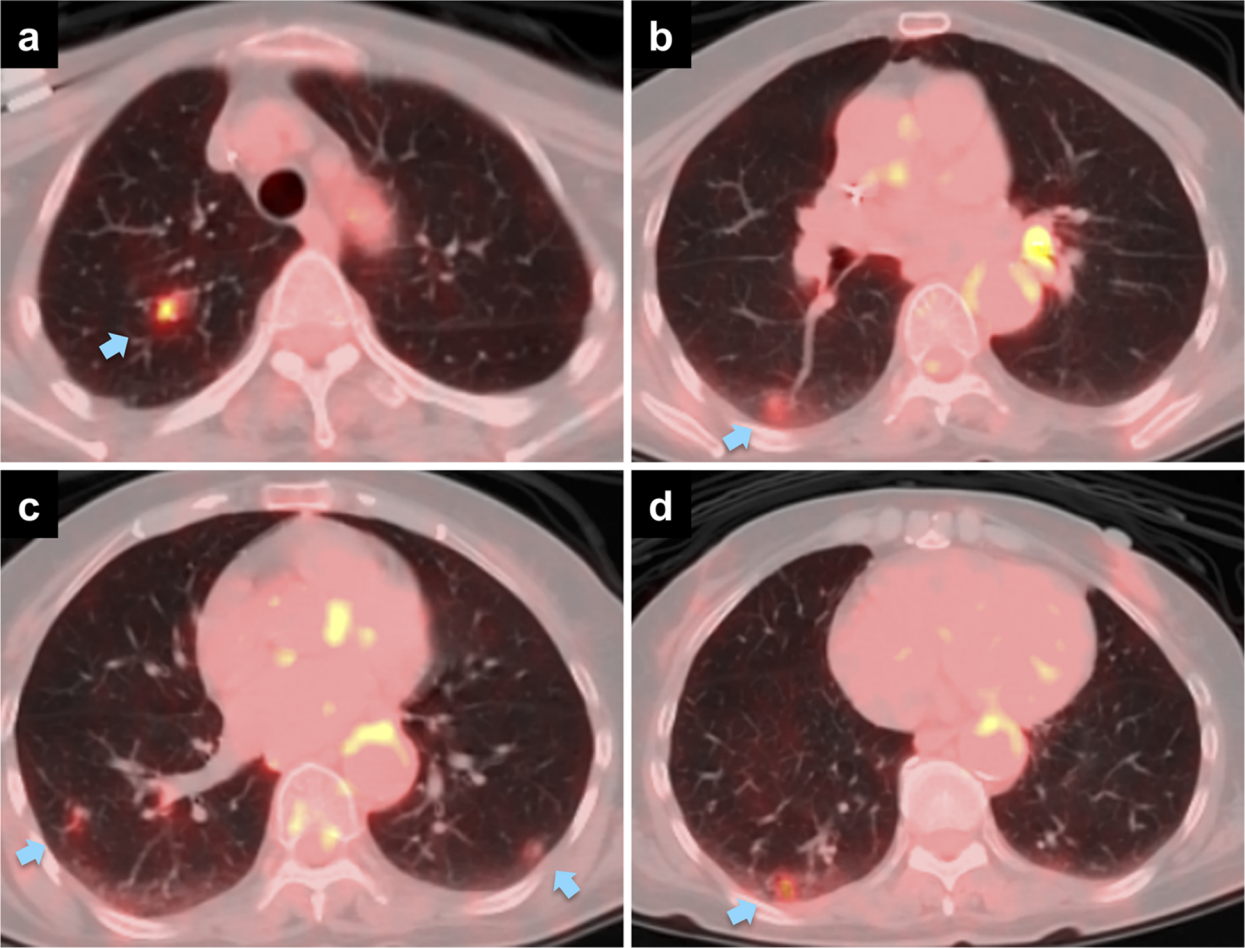
Positron emission tomography integrated with computed tomography scans. ^18^F‐fluorodeoxyglucose accumulated at the (**a**) right S^1^ nodule, (**b**) right S^6^ nodule, (**c**) bilateral S^9^ nodules, and (**d**) right S^10^ nodule (arrows).

A 68‐year‐old, never‐smoking woman with a history of pancreatic cancer and diabetes mellitus was referred for further evaluation of multiple ground‐glass nodules (GGNs) on chest computed tomography (CT). She had been diagnosed with stage III pancreatic cancer and diabetes mellitus seven months before her initial visit to our department. She had undergone chemotherapy with the modified FOLFIRINOX regimen for four months.^7^ One month after the termination of chemotherapy, chest CT showed multiple new GGNs, some of which had pleural tags (Fig 1a,b). After a two month observation period, the GGNs had not improved. Moreover, positron emission tomography showed accumulation of ^18^F‐fluorodeoxyglucose in several GGNs (Fig 2a–d). Lung metastases from pancreatic cancer was suspected.

**Figure 3:**
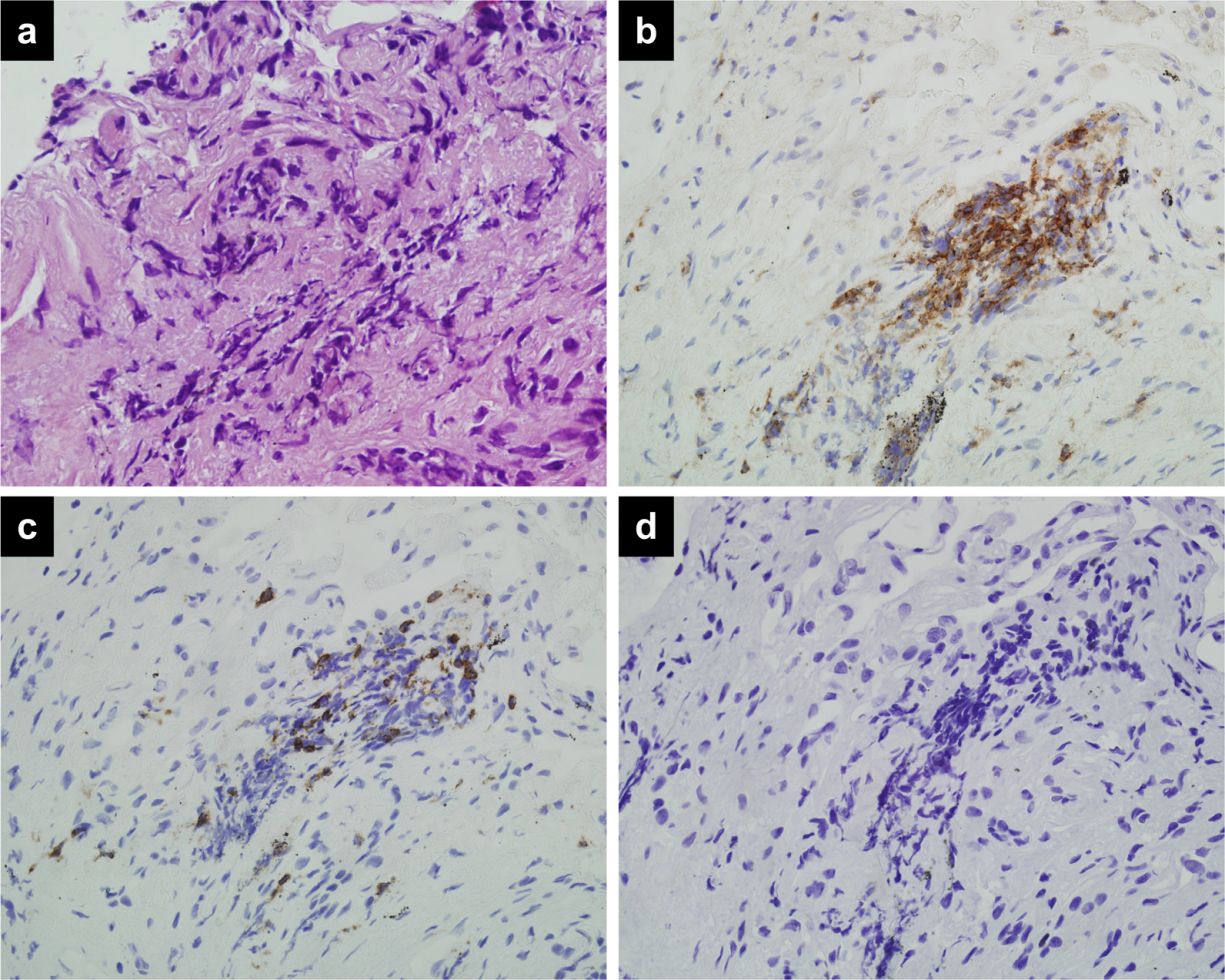
Photomicrographs of transbronchial biopsy specimens (original magnification x40). **(a)** Mild fibrosis and infiltration of lymphocytes in the lung tissue (hematoxylin & eosin stain). Immunohistochemical examination showed (**b**) CD4^+^ cells, (**c**) CD8^+^ cells, and (**d**) FOXP3^+^ regulatory T cells. The antibody clones used were as follows: CD4 (4B12), CD8 (4B11), and FOXP3 (236A/E7).

**Figure 4:**
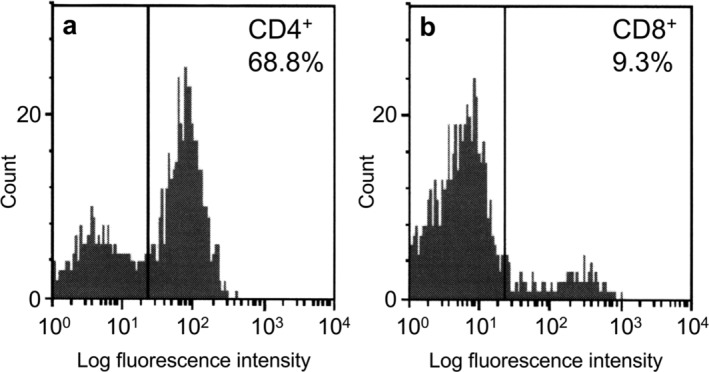
Flow cytometry analysis of bronchoalveolar lavage fluid. (**a**) CD4^+^ cells and (**b**) CD8^+^ cells. The antibody clones used were as follows: CD4 (SK3) and CD8 (SK1).

The patient was asymptomatic and physical examination was unremarkable. On laboratory examination, serum C‐reactive protein and KL‐6 levels were not elevated. Transbronchial biopsy specimens of the right GGNs showed mild fibrosis and infiltration of lymphocytes, but no malignant cells (Fig 3a). Immunohistochemical analysis of the specimens showed that the lymphocytes consisted of more CD4^+^ than CD8^+^ cells (Fig 3b,c). The CD4^+^ cells did not include FOXP3^+^ regulatory T cells (Fig 3d). Bronchoalveolar lavage (BAL) fluid from the right middle lobe confirmed the increase in total cell number (23.5 × 10^4^/mL), lymphocyte composition of 18% with no eosinophils or neutrophils, and a high CD4^+^/CD8^+^ ratio of 7.4 (Fig 4). There was no evidence of other interstitial pneumonia and infectious diseases.

The patient reported that an oral probiotic supplement of *L. paraplantarum* had been taken since the diagnosis of pancreatic cancer along with vildagliptin. A drug‐induced lymphocyte stimulation test, although considered a complementary examination, was 270% for vildagliptin and 560% for the probiotic supplement (reference, <180%). The GGNs disappeared shortly after both agents were discontinued (Fig 1c–f). She was diagnosed with vildagliptin‐induced IP, possibly affected by the *Lactobacillus* probiotic supplement. Subsequently, the IP has not recurred for seven months. Written, informed consent for the publication of this case report was obtained from the patient.

## Discussion

The case reported here provided the following important findings. Chest CT showed multiple GGNs mimicking metastatic lung cancer. The CD4^+^/CD8^+^ ratio in BAL fluid was extremely high, with an increase in CD4^+^ cells, but no regulatory T cells in the lung tissue.

In the present case, vildagliptin‐induced IP showed unusual manifestations mimicking metastatic lung cancer. Several DPP‐4 inhibitors are well known to cause IP.^8^, ^9^, ^10^, ^11^ According to the Japanese Adverse Drug Event Report database, 63 cases of vildagliptin‐related IP were reported between 2009 and 2018.^12^ In the literature, three cases of vildagliptin‐induced IP have been specifically reported.^9^, ^10^, ^11^ On chest CT, diffuse nonsegmental ground‐glass opacities were seen in the above cases，while multiple GGNs were observed in metastatic pancreatic cancer, gastrointestinal cancer, and pulmonary adenocarcinoma cases.^13^ Since probiotic‐induced IP has not yet been reported, the *Lactobacillus* probiotics probably modified the vildagliptin‐induced IP, resulting in the unusual radiological presentation.

The current case showed predominant CD4^+^ cells in the lung tissue and BAL fluid, whereas drug‐induced pneumonia usually shows predominant CD8^+^ cells in BAL fluid as reported by Bonella *et al*.^14^ A low CD4^+^/CD8^+^ ratio in BAL fluid has also been reported in vildagliptin‐induced IP.^10^ Lee *et al*. demonstrated that DPP‐4 was expressed even on T lymphocytes, especially CD4^+^ cells.^15^ DPP‐4 inhibitors have been reported to suppress the proliferation of CD4^+^ cells in vitro.^16^, ^17^ In contrast, the probiotics of several *Lactobacillus* strains were shown to enhance the activity of CD4^+^ Th1 cells in the intestine.^18^ In the study by Morton *et al*. a mouse model demonstrated that intestinal immune cells were capable of migrating to distant organs.^19^ Taken together, the simultaneous intake of *L. paraplantarum* may explain the predominant CD4^+^ cells in the lung tissue and BAL fluid.

The present case report has some limitations. The mechanisms involved in the effects of the probiotics remain undetermined. CD4^+^ cells can functionally differentiate into various subsets which maintain immune homeostasis. For example, regulatory CD4^+^ cells include Egr2^+^ cells, Tr1 cells, and Th3 cells as well as FOXP3^+^ cells.^20^, ^21^ Although CD4^+^ cells did not express FOXP3, the presence of other regulatory CD4^+^ cells was not investigated in the present case. There is a potential mechanism that other regulatory CD4^+^ cells might affect atypical findings of IP on chest CT. Moreover, the effects of the previous chemotherapy were not completely excluded. Nevertheless, the association of the gut microbiota with systemic immune reactions has been attracting attention. The present findings may provide a clue for examining the potential relationship between gut microbiota and the lung immune system.

In conclusion, *Lactobacillus* probiotic supplementation could have modified the clinical presentation of DPP‐4 inhibitor‐induced IP in a cancer patient, leading to a suspicion of metastatic lung cancer. If unusual manifestations of IP are observed in some cancer patients, physicians should take note of the medication history, including probiotic supplements.

## Disclosure

No authors report any conflict of interest.
